# RNAP-II Molecules Participate in the Anchoring of the ORC to rDNA Replication Origins

**DOI:** 10.1371/journal.pone.0053405

**Published:** 2013-01-04

**Authors:** Maria D. Mayan

**Affiliations:** MRC Clinical Sciences Centre, Imperial College, London, United States of America; Instituto Butantan, Laboratório Especial de Toxinologia Aplicada, Brazil

## Abstract

The replication of genomic DNA is limited to a single round per cell cycle. The first component, which recognises and remains bound to origins from recognition until activation and replication elongation, is the origin recognition complex. How origin recognition complex (ORC) proteins remain associated with chromatin throughout the cell cycle is not yet completely understood. Several genome-wide studies have undoubtedly demonstrated that RNA polymerase II (RNAP-II) binding sites overlap with replication origins and with the binding sites of the replication components. RNAP-II is no longer merely associated with transcription elongation. Several reports have demonstrated that RNAP-II molecules affect chromatin structure, transcription, mRNA processing, recombination and DNA repair, among others. Most of these activities have been reported to directly depend on the interaction of proteins with the C-terminal domain (CTD) of RNAP-II. Two-dimensional gels results and ChIP analysis presented herein suggest that stalled RNAP-II molecules bound to the rDNA chromatin participate in the anchoring of ORC proteins to origins during the G1 and S-phases. The results show that in the absence of RNAP-II, Orc1p, Orc2p and Cdc6p do not bind to origins. Moreover, co-immunoprecipitation experiments suggest that Ser2P-CTD and hypophosphorylated RNAP-II interact with Orc1p. In the context of rDNA, cryptic transcription by RNAP-II did not negatively interfere with DNA replication. However, the results indicate that RNAP-II is not necessary to maintain the binding of ORCs to the origins during metaphase. These findings highlight for the first time the potential importance of stalled RNAP-II in the regulation of DNA replication.

## Introduction

Replication originates at multiple and specific locations in the genome that define starting sites, which are called replication origins (ORIs) [1]. In the case of mammalian cells, replication origins have predominantly been found to be located in promoter regions [2], [3], [4], [5], [6] **.** In contrast, in *S. cerevisiae*, ORIs have been found in restricted regions that are referred as autonomously replicating sequences (ARSs) [7], [8]. The initiation of chromosomal DNA replication is a complex process that assures that every replication origin fires once and only once during the S-phase. The initiation, activation and firing are tightly linked but have their own characteristics. The primary principles of the replication initiation are conserved from yeasts to humans [9], [10]. Origin selection and activation occurs in three steps. (a) The origins are recognised by the pre-replication complex (pre-RC), which is a process called licensing and is responsible for recruiting additional replication factors to this site. The pre-RC typically forms at the end of mitosis by the binding of origin recognition complex (ORC) [11] to chromosomal DNA, which enables the interaction of the next replication proteins, which are Cdc6, Cdt1 and six MCM subunits (Mcm2–7). However, the manner in which the loading of all of these components is precisely regulated remains poorly understood. Pre-RC formation renders the chromatin competent for replication and is restricted to the G1 phase of the cell cycle, forming the so-called “window of opportunity”. (b) The second step in the triggering of origin activation is the recruitment of additional initiator proteins to the pre-RC to form the pre-initiation complex (pre-IC). (c) Finally, DNA synthesis initiates after an activation step.

A report demonstrated that in HeLa cells, the ORC is localised to specific sites that frequently overlap with RNA polymerase II-associated sequences [**3**]. These authors and others suggest that the elements bound to the RNA polymerase II-associated sequences may participate in the activation of the origins [2], [3], [12]. RNA polymerase II (RNAP-II) molecules have been found bound to chromatin although their genes were not expressed in a phenomenon that has been called “poised or stalled RNAP-II” [13]. The C-terminal domain (CTD) of the largest subunit of RNAP-II (Rpb1p) contains multiple repeats of the heptapeptide Tyr-Ser-Pro-Thr-Ser-Pro-Ser [14], [15]. Modifications such as the phosphorylation of the serine residues of the CTD have been reported to be responsible for the recruitment of transcription factors and other proteins to the chromatin [16], [17], [18]. In yeast, the ARS1 consensus sequence contains a binding site for the transcription factor Abf1p [19], and it has been shown that the recruitment of transcription factors to ARS1 is sufficient to enhance replication from a minichromosome origin [20], [21]. Intriguingly, it has been reported that the CTD of the RNAP-II complex binds to and likely regulates the activity of ARS1 [22]. Moreover, in general, the temporal firing order often correlates with transcriptional activity; early-replicating regions of chromosomes are associated with active genes, and late-replicating regions are associated with silent genes [23], [24].

In virus, yeast, *Drosophila*, Xenopus and mammalian cells, transcriptional elements at replication initiation sites have been shown to stimulate replication [19], [20], [21], [25], [26], [27]. It has been suggested that this stimulation may be a consequence of direct interaction with components of the pre-RC [28]. However, the majority of published reports have concentrated on the study of the accessibility of the replication components to DNA depending on the chromatin structure or on the effect of transcription [29], [30], [31]. Why and how ORIs locate at the RNAP-II binding sites is not yet entirely understood. RNAP-II molecules, through the recruitment of several proteins, are able to modify the transcriptional firing, mRNA processing and chromatin structure of DNA sequences to which they are bound [16], [18], [32], [33], [34]. The results presented here are the first evidence to suggest that RNAP-II molecules may directly participate in the anchoring of the pre-replication complex to the rDNA locus. The results suggest that the binding of the replication proteins Orc1p, Orc2p and Cdc6p to the ORIs at the yeast rDNA locus occurs via RNAP-II independent of transcription elongation but requires chromatin-bound stalled RNAP-II molecules. Furthermore, the binding of Orc1p and Orc2p to ARS607 and ARS1412, which are an early and late ORIs, respectively, was also dependent on the presence of RNAP-II complexes.

## Results

### The Absence of RNAP-II Complexes Alters Replication at the rDNA Locus

**Figure 1:**
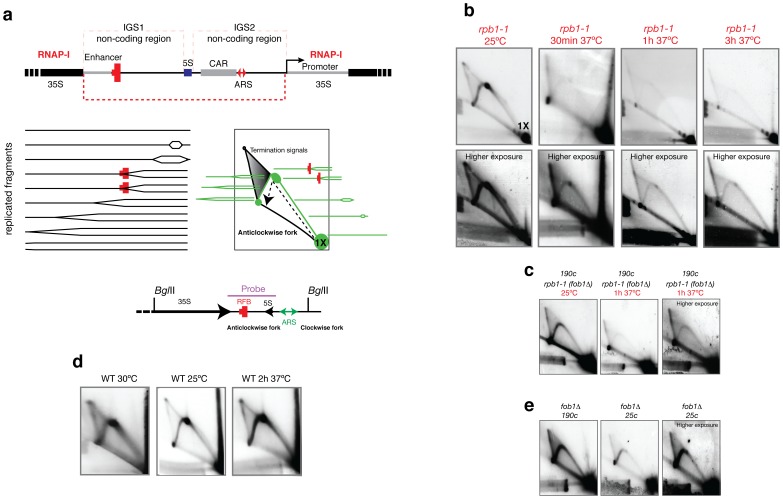
The replication of rDNA is affected in the absence of the largest subunit of RNAP-II. (**a**) Diagram of the rDNA with the locations of the replication barrier (RFB), replication origin (ARS) and cohesin binding sequences (CAR). Below, the theoretical schemes for the two-dimensional agarose gel electrophoresis of chromatin digested with *Bgl*II are depicted. The accumulation of RIs at the RFB is expected at 1.49X. 1X represents the position of the linear, unreplicated fragment. The hybridisation probe used for the Southern protocol and hybridisation was amplified by PCR and column purified (Qiagen). The amplified fragment is represented above**.** The direction of the anticlockwise and clockwise replication fork is shown below. (**b**) Results obtained after the isolation of DNA from asynchronous cultures growing exponentially at different temperatures. Higher exposure images are shown below. Significantly fewer amounts of RIs were detected in the absence of *RPB1* (*rpb1–1 ts* strain). 1X signals were similar among the 4 experiments (25°C, 37°C for 30 minutes, 37°C for 1 hour and 37°C for 3 hours). The same number of cells was analysed. (**c**) Results obtained after *fob1* deletion in the *rpb1–1 ts* strain. (**d**) To control the effect of the temperature on DNA replication, a wild type strain (BY4741) was used. (**e**) Results obtained after the isolation of DNA from a *fob1*Δ strain containing approximately 190 or 25 rDNA copies. A higher exposure image is shown on the right for the 25 copies strain.

The ribosomal DNA of *S. cerevisiae* consists of several hundred repeats that contain sequences encoding the *35S* and *5S rRNA* genes, which are separated by two intergenic non-coding regions (IGS1 and IGS2) (Figure 1a). Every repeat contains a replication origin, which is located in the IGS2. A polar replication fork barrier (RFB) is located the adjacent intergenic region that ensures the unidirectional replication of the locus, likely to prevent collision between the 35S transcription and the moving replication forks (Figure 1a) [35], [36]. RNAP-II transcribes and binds to IGS1 and IGS2 (ARS) [37], [38]. However, the polymerase is primarily found in a stalled ternary complex [38]. In the present study, RNAP-II was also found bound to the ARS located in the intergenic region of the *S. Pombe* rDNA locus and close to the origin in mammals (Figure S1a).

To study the effect of the absence of RNAP-II complexes in the replication of the rDNA locus, a conditional allele of the biggest RNAP-II subunit, RPB1, which becomes degraded when the mutant cells are exposed to high temperature, was used (Figure 1a, 1b and 1c. See Figure S2a). To test the replication activity, neutral-neutral two-dimensional electrophoresis (2D gels) was used to investigate the presence of replication intermediates (RIs) (Figure 1a). When the cells where grown at 25°C and DNA was digested with *Bgl-*II, 2D gels showed the typical simple Y pattern showing the forks stalled at the RFB sequence (spot signal) with a mass of 1.45 times the mass of the linear unreplicated form (Figure 1a and 1b). These forks start replication at the origin located at the IGS2. When the anticlockwise fork reaches the RFB in IGS1, it is paused or stalled at that site in the presence of the Fob1p (Figure 1a and 1b). Some of these forks are able to cross the barrier, as shown by the presence of RIs signals after the site. The presence of more replication intermediates after the RFB than before indicates that when the replication initiates, the forks move quickly, but after they reach the RFB, the forks are slowly released, as indicated by the accumulation of RIs. Another explanation is the presence of transcription in the *35S rRNA* gene, which may collide with the anticlockwise forks and slowing the velocity of the forks.

Surprisingly, the loss of RNAP-II complexes by shifting the cells to 37°C provoked the loss of RIs (Figure 1b and c). The loss of RIs is a drastic event, which occurred only after 30 minutes at 37°C. After overexposure, some RIs were still observed, suggesting that some replication forks were still elongating after 3 hours at 37°C. As a control, a wild type strain, BY4741, was grown at 25°C and 37°C for 2 hours. Higher levels of RIs were detected at the higher temperature (Figure 1d). To control the number of active ORIs, two strains (NOY1064) with different rDNA copy numbers were used [39] (Figure 1e). In both strains, *fob1* was deleted, and therefore, the RFB was not active. Figure 1c shows the RIs detected in strain Z118 (*rpb1–1 ts*) with *fob1* absent. The strain with 25 copies showed fewer RIs than the strain with 190 copies (Figure 1e), and the higher exposure (25 copies) showed much higher levels of RIs than the higher exposure of Z118 when RNAP-II was degraded (Figure 1c and 1e).

In the absence of RNAP-II, the 1.49X signal (RFB) showed similar levels to the remaining RIs, implying that the presence of simple Y arcs in these gels likely indicates passive replication of the studied DNA fragment (Figure 1b). In fact, the loss of the bubble arc but not the arc of simple Y arcs after digesting the DNA with *Stu*I confirms that the RIs detected in the studied fragment likely corresponded to passive replication by forks that had previously initiated replication in origins located elsewhere (Figure S2). However, comparing Figure 1e (25 copies) to 1c (Z118 (*rpb1–1)*, ∼200 copies) (higher exposure) indicates the presence of few RIs in *rpb1–1* at 37°C. Together with the total loss of the bubble arc (Figure S2c), the results suggest that RNAP-II may affect the pre-RC or the pre-IC more than the DNA synthesis.

### Cryptic Transcription by RNAP-II is Not Required for DNA Teplication at the Ribosomal Locus

**Figure 2:**
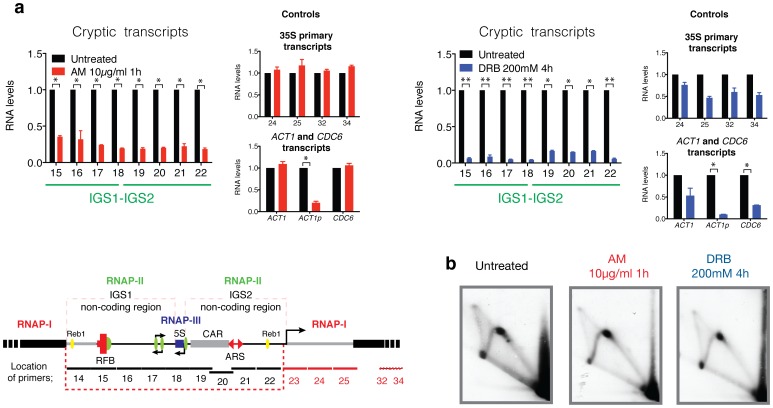
The inhibition of RNAP-II does not alter the replication of rDNA. (**a**) *rpb1–1 ts* strain grown at 25°C. Exponentially growing cultures were treated with 10 µg/ml of AM for 1 hour or with 200 mM of DRB for 4 hours before cell harvesting as previously reported [44]. The quantification of RNA was performed using real-time qRT-PCR. The RNA levels are shown relative to each primer (untreated). To compare the conditions, the data were normalised relative to the mature form of the U2 small nuclear RNA [38]. A representative diagram of the rDNA gene locus on chromosome XII is shown below. Specialised features of the IGS include the RNAP-II promoter (green circles), cohesin binding sequence (CAR) (grey box), a replication origin (ARS) in IGS2 and a replication fork barrier (RFB) in IGS1. The locations of the primers used in this study are shown. All values are expressed as the mean ± S.E.M. n = 2, n = 3 or n = 4. **p<0.005, *p<0.05 for Student’s t*-*test, untreated versus treated. (**b**) 2D gels of the RIs corresponding to untreated and AM and DRB treated samples.

To study the effect of cryptic transcripts in rDNA replication, two inhibitors of RNAP-II transcription were employed (Figure 2). α-amanitin (AM) inhibits the translocation step of the transcript polymerisation reaction, promoting the degradation of the elongating polymerase [40], [41], [42], [43]. This process enables study of the effect of blocking RNAP-II transcription without preventing the binding of RNAP-II ternary complexes that are stalled on DNA because RNAP-II sensitivity to AM depends on elongation. The other inhibitor studied, 5,6-dichloro-1-beta-d-ribobenzimidazole (DRB), inhibits CDK9-dependent S2 phosphorylation and therefore transcription elongation. Both inhibitors prevent the elongation of processive RNAP-II complexes by a different mechanism. Permeabilised cells were treated with 10 µg/ml of AM for 1 h and with 200 mM of DRB for 4 hours [44]. The levels of cryptic transcripts were quantified by Real Time PCR and are shown in Figure 2a. The RNAP-II-cryptic transcripts and the *ACT1* primary transcripts decreased significantly, but the total mRNA levels of *ACT1* and *CDC6* did not significantly change after 1 hour of treatment. RNAP-I transcription was not affected using this concentration of AM (see the 35S primary transcripts analysed). Instead, the concentration used for DRB treatment affected the transcription RNAP-I [45], [46]. In fact, after 4 hours of treatment, all of the RNA species had decreased. Under these conditions, it is expected that cells have less proteins available. However, even after 4 hours of transcription inhibition by RNAP-II, RIs were still detected in 2D gels (Figure 2b).

### Stalled RNAP-II Molecules Participate in the Binding of the ORC to ARSs

**Figure 3:**
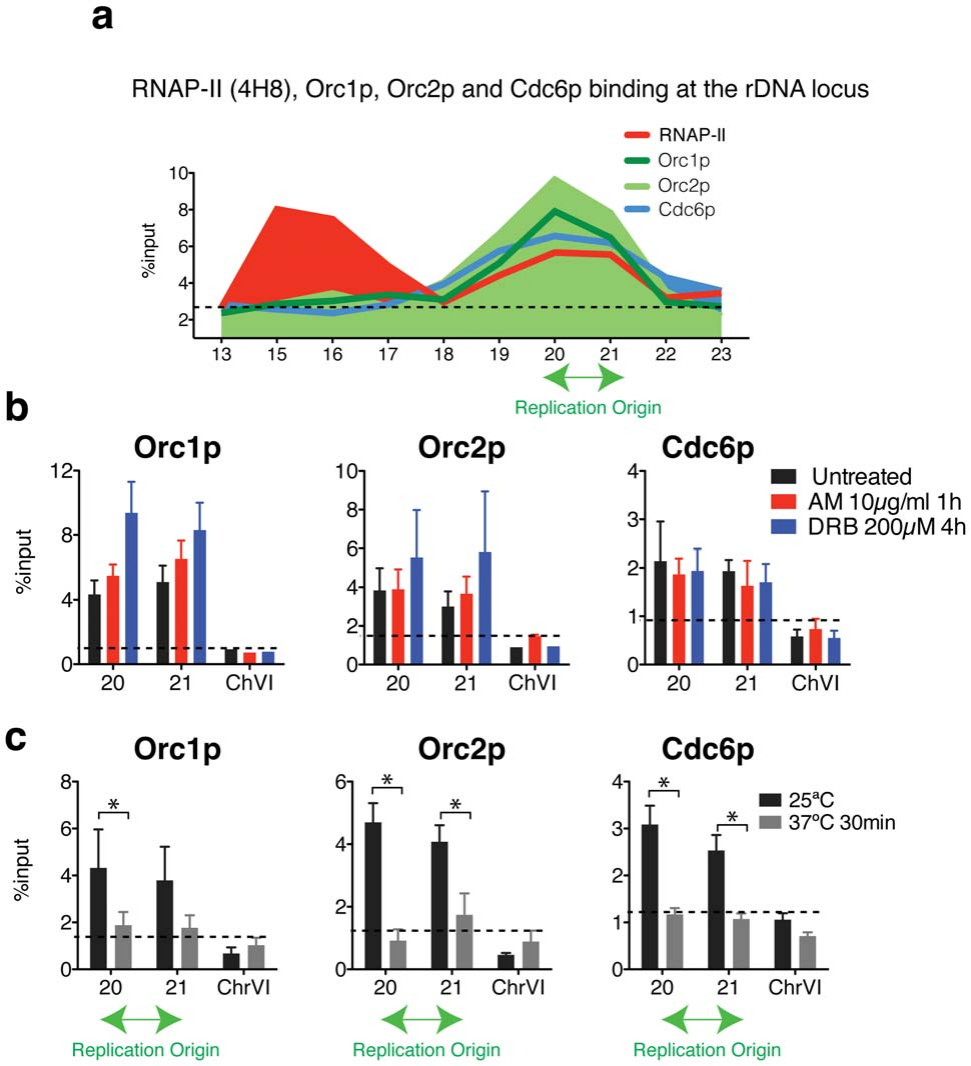
The inhibition of transcription by RNAP-II does not affect the binding of replication components to the rDNA locus. (**a**) Chromatin immunoprecipitation (ChIP) analysis of RNAP-II (4H8), Orc1p, Orc2p and Cdc6p binding within the intergenic spacers (primers 15 to 22), *35S* (primer 13 and 23) and *5S* gene regions (primer 18) in wild type cells. Orc1p, Orc2p and Cdc6p bound to cohesin (CAR, primer 19 and 20) and ARSs (p20, p21). (**b**) ChIP analysis of Orc1p, Orc2p and Cdc6p bound to the ARS in a *rpb1–1 ts* strain growing at 25°C. The results were obtained for untreated and cells that were treated with AM for 1 hour or DRB for 4 hours. All values are expressed as the mean ± S.E.M. n = 2 or n = 3. **p<0.005, *p<0.05 for the Student’s t*-*test, untreated versus treated. (**c**) ChIP analysis of the *rpb1–1 ts* strain growing at 25°C or shifted at 37°C for 30 minutes. Mean ± S.E.M. n = 3 or n = 4. **p<0.005, *p<0.05 for Student’s t*-*test, 25°C versus 37°C. A sequence located in chromosome VI was used as a negative control (background) for the binding of the replication proteins.

ChIP experiments were used to determine the binding of ORC elements to ARSs (Figure 3a). Whereas RNAP-II binds to the IGS1 and IGS2 regions (red line), Orc1p, Orc2p and Cdc6p were only localised in the ARS1 sequence. Orc1p is the largest subunit of the ORC that is required for DNA binding and is involved in the formation of pre-RCs. Orc2p, in contrast, forms part of the core complex and interacts with Cdc6p, which is involved in the assembly and the maintenance of pre-RCs. ChIP analysis showed that the inhibition of RNAP-II transcription using AM and DRB treatments does not alter the binding of Orc1p, Orc2p and Cdc6p to the origins (Figure 3b). After both treatments, transcription elongation is inhibited, but stalled RNAP-II molecules remain bound to the chromatin [38]. To test if the CTD of RNAP-II was implicated in the interaction of the ORC with ARS, ChIP was employed to study the binding of Orc1p, Orc2p and Cdc6p in the absence of *RPB1* (*rbp1–1* strain at 37°C). After only 30 minutes at 37°C, Orc1p, Orc2p and Cdc6p binding to the ARS significantly decreased. The results are shown in Figure 3c.

### RNAP-II Protects Orc1p, Orc2p and Cdc6p Binding to the Chromatin during the G1 and S-phases

**Figure 4:**
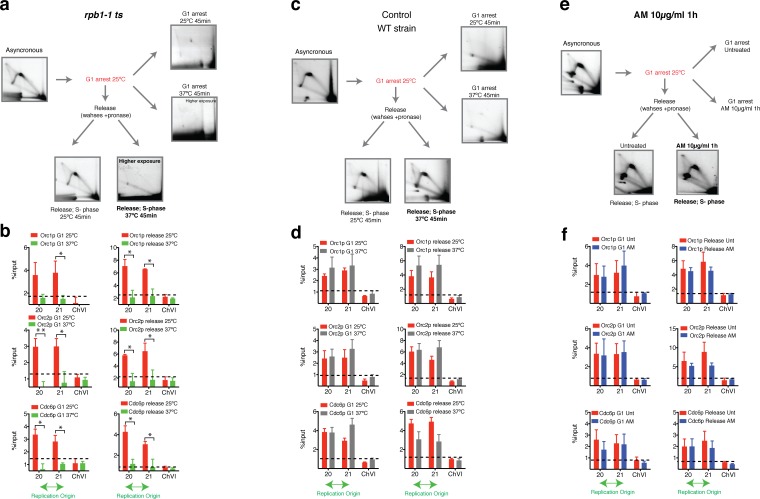
RNAP-II participates in the anchoring of Orc1p, Orc2p and Cdc6p to the ARS. (**a**) 2D gel of the RIs corresponding to an asynchronous culture (*rpb1–1* strain) that was arrested with α-factor in G1 at 25°C and subsequently was divided into four aliquots. α-factor at 25°C and α-factor at 37°C for 45 additional minutes: Two flasks were released with pronase, and the cells were grown at 25°C or shifted to 37°C for 45 minutes. (**b**) ChIP analysis of Orc1p, Orc2p and Cdc6p within the IGS regions in *rpb1–1* cells arrested in G1 at 25°C and shifted to 37°C. On the right is the ChIP analysis obtained after releasing cells 45 minutes after pronase at 25°C or 37°C. (**c**) and (**d**) ChIP analysis and images corresponding to a 2D gel obtained from an asynchronous culture (wild type) that was arrested in G1 with α-factor and subsequently divided into four aliquots as described above. (**e**) and (**f**) ChIP analysis and 2D gels show the RIs of the asynchronous culture (wild type) used to arrest cells in G1 (α-factor). The cells were incubated for 45 additional minutes in the presence or absence of 10 µg/ml of AM. The cells that were released from α-factor were incubated in the presence or absence of AM. The fold enrichment relative to a sequence located in ChVI is shown. Mean ± S.E.M. n = 2 or n = 3. **p<0.005, *p<0.05 for Student’s t*-*test, 25°C versus 37°C, untreated versus treated.

It has been shown that initiations sites are selected anew in each cell cycle by an unknown mechanism at the origin decision point. The results in Figure 3 were obtained with exponentially growing asynchronous cultures. To better understand the effect of RNAP-II on pre-RCs formation, the cells were synchronised in G1 using alpha-factor (Figure 4). An exponentially growing asynchronous culture was synchronised in G1. When 99% of the cells were in G1 phase, which was confirmed with microscopy by the appearance of the typical single form, the culture was split in two. One flask was maintained for 45 minutes at 25°C, and the other flask was maintained at 37°C for 45 minutes (Figure 4a, G1 arrest). Note that G1 arrested cells used for the release experiments were not shifted to 37°C during the arrest or treated with AM before release, to allow the transcription of specific proteins implicated in cell cycle progression. The cells were treated with AM or shifted to 37°C when pronase, to start the release, was added to the culture. In contrast, for the G1 arrest experiments, cells arrested in G1 were treated with AM or shifted to 37°C for 45 minutes in the presence of alpha-factor. The schematic representations of the experiments are shown in figure 4.

Using 2D gels, RIs were not detected in G1 phase. Using ChIPs to study the formation of the pre-RC, Orc1p, Orc2p and Cdc6p binding was detected during G1 (Figure 4b). However, at 37°C in the absence of RNAP-II, the binding of Orc1p, Orc2p and Cdc6p decreased significantly (Figure 4b). Arrested G1 cells at 25°C were washed with pronase to degrade the alpha-factor and release the cells to S-phase at 25°C or at 37°C for 45 minutes. Tiny buds were detected under the light microscope at 25°C. In contrast, at 37°C, the cells were never released. The binding of Orc1p, Orc2p and Cdc6p decreased significantly compared with the binding in the culture at 25°C (Figure 4b). These results were confirmed by 2D-gels. After the release at 37°C, cells probably failed to enter in S-phase and in fact RIs were no longer detected, even at higher exposure (Figure 4a). These results suggest that the RIs found at higher exposure in an asynchronous culture (Figure 1b and c) likely belong to the replication forks of the origins that were activated before the degradation of RNAP-II occurred. The wild type strain BY4741 was used as a control for the temperature shift. The same experiments did not show any significant change in the binding of pre-RC proteins to ORIs when the wild type cells were grown at 37°C (Figure 4 c and d). The binding of Orc1p, Orc2p and Cdc6p did not change after 1 hour of treatment with AM, and the cells released perfectly well from G1 to S-phase (Figure 4e and 4f).

### Orc1p Interacts with RNAP-II Complexes Containing Hypophosphorylated and Ser2P-RNAP-II Molecules

**Figure 5:**
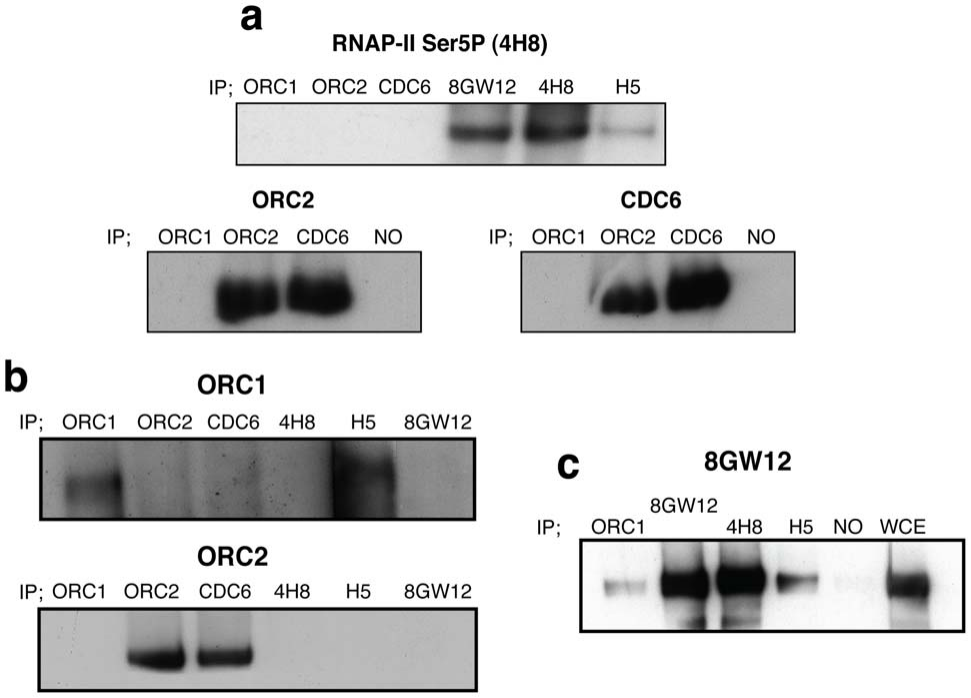
Co-immunoprecipitation experiments suggest that RNAP-II complexes interact with Orc1p. (**a**) Western-blot analysis using 4H8 antibody for immunoprecipitated Orc1p, Orc2p, Cdc6p and RNAPII (using the 4H8, 8GW16 or H5 antibody). Hypophosphorylated (8GW16) and Ser2P-CTD (H5) phosphorylated at Ser5 (4H8). The same number of cells was used for each immunoprecipitation experiments (see materials and methods). Western-blot analysis using Orc2p or Cdc6p antibody, showed the interaction between Orc2p and Cdc6p. NO (no antibody) (**b**) Western-blot using Orc1p antibody for immunoprecipitation revealed that Ser2P-RNAP-II (H5) interacts with Orc1p. No interaction of RNAP-II with Orc2p was detected when Orc2p antibody was used for the western-blot (**c**) Co-IP results show that hypophosphorylated RNAP-II complexes (8GW16) interact with Orc1p, 4H8 and H5. The results for whole cell extraction (WCE) and immunoprecipitation performed without antibody (NO) are shown.

The results herein presented did not demonstrate if the RNAP-II is directly responsible for the maintenance of ORC binding to origins. Co-IP experiments have not shown any interaction between the typically described form of stalled RNAP-II phosphorylated in the Ser5 (4H8) with Orc1p, Orc2p or Cdc6p (Figure 5a). The RNAP-II molecules recognised by the 4H8 antibody recognise or interact with other ternary complexes of RNAP-II. Ser2P-RNAP-II is recognised by the H5 antibody, and the hypophosphorylated RNAP-II molecules are preferentially recognised by 8GW16 antibody [47], [48], [49]. As previously described and shown in Figure 5a (below), Orc2p and Cdc6p interact. However, Orc1p and Orc2p form part of the ORC bound to the origins, and these experiments did not show interaction between Orc1p and Orc2p. Therefore, it is possible that stalled RNAP-II phosphorylated at the Ser5 could be implicated in the recruitment of the ORC through other proteins or through a labile interaction. Moreover, an interaction between the RNAP-II phosphorylated on the Ser2 (H5) and Orc1p but not with Orc2p (Figure 5b) was detected. The results shown in Figure 5c also suggest an interaction between hypophosphorylated RNAP-II and Orc1p. Ser5 residues and the CTD retain their recognition by 8GW16.

### Stalled Ser5P and Ser2P-RNAP-II Complexes at the rDNA Locus Favor Replication

**Figure 6:**
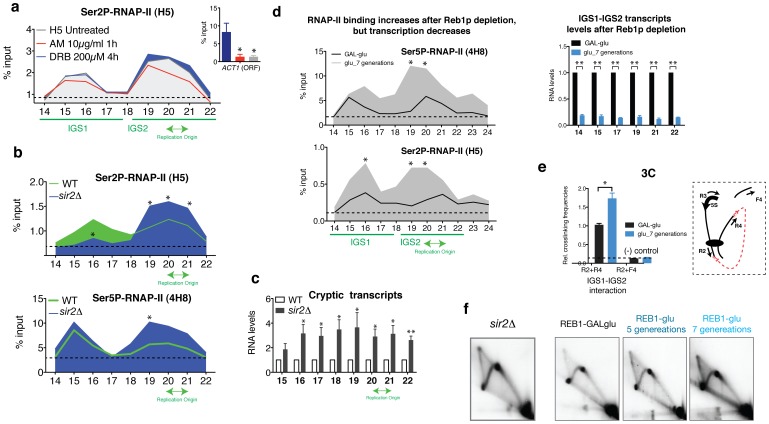
The levels of RNAP-II bound to the chromatin affects DNA replication at the rDNA locus. (**a**) ChIP analysis shows the binding of Ser2P-CTD to the rDNA before and after treatment with AM and DRB. Values are expressed as the mean±S.E.M. n = 2. (**b**) ChIP analysis of Ser2P-CTD (H5) and Ser5P-CTD (4H8) using a wild type strain and *sir2Δ* within rDNA IGS regions. Mean ± S.E.M. n = 3. *p<0.05 for Student’s t*-*test, wild type versus *sir2Δ*. (**c**) rtPCR-based analysis of transcripts within IGS regions in wild-type and *sir2Δ.* Mean ± S.E.M. n = 3. **p<0.005, *p<0.05 for Student’s t*-*test, wild type versus *sir2Δ.* (**d**) ChIP analysis of RNAP-II (4H8 and H5) binding in a *GAL-REB1* strain. The cells were grown in a 2% galactose/0.3% glucose medium (+REB1) and shifted to 2% glucose to repress *REB1* expression (REB1-). An RT-PCR-based analysis of the IGS transcripts is shown on the right. Mean ± S.E.M. n = 2 o n = 3. **p<0.005, *p<0.05 for Student’s t*-*test, REB+ versus REB- (7 generations). (**e**) 3C analysis in a *GAL-REB1* strain. Controls for random ligation (R2+F4; [38]) are shown. Mean ± S.E.M. n = 3. *p<0.05 for Student’s t*-*test, REB1+ versus REB1-. (**f**) 2D gels of RIs corresponding to *sir2Δ* and GAL-REB1 strain grown in a 2% galactose/0.3% glucose medium (REB1+) or in 2% glucose for 5 or 7 generations (REB-).

Together, the ChIPs and the co-immunoprecipitation experiments suggest that stalled RNAP-II complexes containing at least RNAP-II phosphorylated in Ser2 may be involved in the recruitment or binding of Orc1p to the replication origin. ChIP analyses after treating the cells with AM and DRB confirmed that stalled complexes may also include Ser2P-RNAP-II molecules. Ser2P-CTD remained bound to both IGS1 and IGS2 regions after transcription inhibition (Figure 6a). The binding pattern of Ser2P differed slightly from the binding pattern of stalled Ser5P (4H8) that has been previously reported (Figure 6b, wild type 4H8) [38]. Ser2P-RNAP-II molecules (H5 antibody) bound preferentially to IGS2, where the ARS is found (Figure 6a).

For the *sir2*Δ strain, which has been reported to trigger more ORIs [50], the binding pattern of Ser2P-RNAP-II changed drastically compared to a wild type strain (Figure 6b). Ser2P-RNAP-II binding increased specifically near the ARS element (Figure 6b). The binding pattern of 4H8 did not change, but its binding also increased in the IGS2 region (ARS). For both cases, the increase was statistically significant for the cohesin binding site (P19 primer) and for the ARS (primer 20 and 21) in the case of Ser2P. In contrast, as expected, the *sir2*Δ strain showed higher levels of RNAP-II-cryptic transcripts [51] (Figure 6c), indicating that cryptic transcription by RNAP-II does not disturb the activity of the ORIs in ribosomal DNA (2D gel, Figure 6f).

The positive effect of the stalled RNAP-II ternary complex on DNA replication was confirmed using the GAL-REB1 strain. We have previously reported that stalled RNAP-II complexes mediate the chromatin interaction between IGS1 and IGS2 [38]. The results from the present study reveal that Reb1p promotes the activity of stalled RNAP-II molecules in rDNA by promoting its effect on the DNA interaction between IGS1 and IGS2 (Figure 6e). When cells were grown in glucose, REB1 expression gradually decreases after 5 to 7 generations [38]. ChIP analysis of H5 and 4H8 antibodies showed that, in the absence of Reb1p, RNAP-II molecules were strongly recruited to the IGS1-IGS2 regions of the rDNA (Figure 6d). 3C experiments showed that IGS1-IGS2 interaction increased significantly (Figure 6e). However, RNAP-II-cryptic transcripts, which were detected by real-time PCR, decreased significantly (Figure 6d). Under these conditions, where the cryptic transcription decreased but RNAP-II molecules remained bound to the rDNA locus, 2D gels indicate that cells continued to replicate in the absence of both Reb1p and cryptic transcripts (Figure 6f).

### RNAP-II Molecules Maintain the Binding of ORCs to ARS Core Consensus Sequences Located on Different Chromosomes

**Figure 7:**
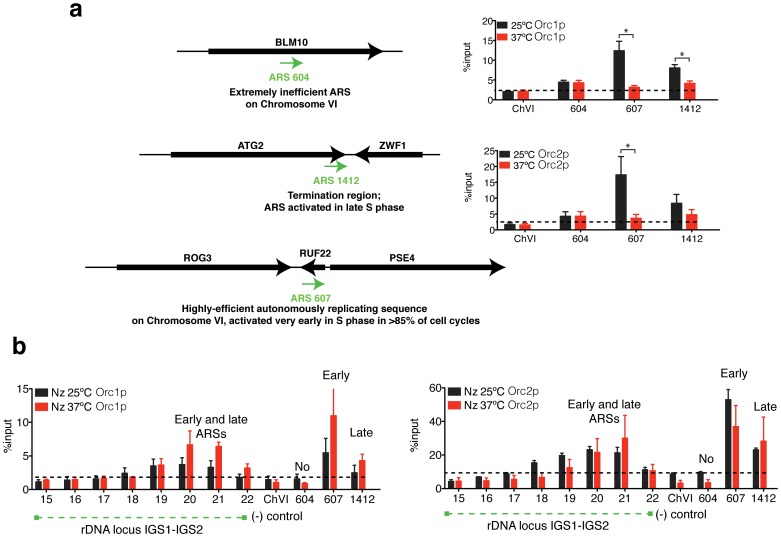
Rpb1p does not participate in the binding of ORC complex to replication origins in nocodazole arrest. (**a**) Schematic representation of the ORIs located at different chromosomes. ChIP analysis of Orc1p and Orc2p to ARS604, ARS1412 and ARS607 in *rpb1–1* strain at 25°C and shifted at 37°C for 45 minutes. Mean ± S.E.M. n = 3. *p<0.05 for Student’s t*-*test, 25°C versus 37°C. (**b**) ChIP analysis of Orc1p and Orc2p within the rDNA IGS regions of chromosome XII and ARSs located in different chromosomes in *rpb1–1* cells arrested in metaphase at 25°C (with nocodazole) and shifted to 37°C for 45 additional minutes. A chromosome VI sequence was used as a negative control for the binding of the replication proteins. Mean ± S.E.M. n = 3.

To test if RNAP-II participates in the recruitment of ORCs to other origins located in different chromosomes, three different ORIs were selected. ARS604 lies within a transcribed gene that is inactive for replication initiation. ARS607 is located in a promoter region and is triggered in early S-Phase, and ARS1412 which is located at the termination site of a coding region and fires in late S-Phase (Figure 7a). The three examples include the replication activity of the majority of the ORIs in yeast [52]. ChIPs results using the *rpb1–1 ts* strain showed that in the absence of RNAP-II (37°C), the early and late ORIs (ARS607 and ARS1412, respectively) showed less binding of Orc1p and Orc2p, but Orc1p and Orc2p binding did not change for the extremely inefficient origin located in the ORF of the *blm10* gene (ARS604) (Figure 7a).

### ORCs Stay Bound to ARS Elements during Mitotic Arrest Independent of the Presence of RNAP-II

To test if stalled RNAP-II molecules are also necessary for the anchoring of ORCs to the origins during mitosis, cells were arrested in metaphase using nocadozole. When 99% of cells were arrested, the culture was split, and half of the mitotic arrested culture was shifted at 37°C for 1 hour to deplete the largest subunit of RNAP-II (Figure 7b). The other half (control) was kept at 25°C. After 1 hour at 37°C, in the absence of RNAP-II, ORC remained bound to the ORIs in the rDNA and to the ORIs located in different chromosomes. No statically significant difference in the binding of Orc1p and Orc2p at 25°C and 37°C was found. However, Orc1p binding tended to increase in the absence of RPB1.

## Discussion

The aim of this project was to study whether RNAP-II influences the activity of the ORIs in the rDNA locus of *S. cerevisiae*. We have previously reported that stalled RNAP-II complexes at the rDNA locus contain the typical form of stalled RNAP-II phosphorylated in the residue Ser5 of its CTD [38]. However, as it has been previously observed at other DNA sequences [53], stalled RNAP-II complexes bound at the rDNA locus likely contain other CTD modifications, such as Ser2P-CTD (Figure 6a). Moreover, ChIP analysis has revealed that RNAP-II complexes bound to the rDNA locus contain Ser7P-CTD and hypophosphorylated RNAP-II molecules (Figure S3). Different studies have demonstrated that the CTD of RNAP-II interacts with several replication components, such as MCMs and DNA polymerase ε [54], [55]. However, the presence of DNA repair, replication or recombination factors in purified CTD-RNAP-II has been found to depend strongly on the purification strategy employed [54], [56], [57]. The results presented herein suggest that hypophosphorylated RNAP-II and Ser2P-CTD RNAP-II complexes, but not Ser5P-CTD, interact with Orc1p (Figure 5). The downregulation of cryptic transcription by RNAP-II did not affect ORC binding to the chromatin or DNA replication studied by 2D gels. However, the origins that lost the RNAP-II bound at the intergenic regions also lost the ORC and likely became inactive. ChIP-chip experiments have demonstrated that RNAP-II stalling is a genome-wide phenomenon that is more common than previously thought 13,58,59. Whereas this report suggests that RNAP-II complexes participate in the binding of ORCs to the origins, other reports have suggested that transcription factors such as c-myc or c-jun may act as potential regulators of origin selection [3], [60], [61], [62]. To date, neither the results presented here nor the results obtained by others can elucidate whether RNAP-II molecules bound to the chromatin or other transcription factors via interaction with RNAP-II complexes are responsible for the origin activity. Specific combinations of transcription factors together with the largest subunit of RNAP-II (*RPB1*) are likely implicated in origin activation, usage and transcription regulation, depending of the cell cycle (See Figure S4). In fact, transcription factors at origins have been shown to stimulate or inhibit replication initiation [26]. Additionally, ORCs have been shown to function as negative regulators of transcription in yeasts and mammals [63], [64], [65], [66], [67]. Moreover, binding sites for Rap1p and Abf1p, which stimulate replication initiation at some ARSs [19], contribute to ARS-mediated transcriptional silencing [68], [69]. Interestingly, the ChIP analysis shown in Figure S1b suggests that the binding of Rap1p to the rDNA locus depends on RPB1. On the other hand, the different modifications in the C-terminal heptad repeat Domain (CTD), have been implicated in the recruitment of factors that modulate chromatin state and transcription [14]. Consequently, the lack of stalled RNAP-II complexes attached to IGSs regions may also affect the structure of chromatin and therefore the recruitment of proteins to the ARS sequence. Other indirect mechanisms affecting origin activity cannot be ruled out. However, results reported here together with results previously published suggest that stalled RNAP-II complexes bound to origins are crucial to maintain the ORC complex bound to the origin (Figure S4). Alternatively the CTD of RNAP-II may also recruit other factors which are important for origin function.

It has been reported that some RNAP-II ternary complexes stay engaged throughout mitosis [70], [71], [72]. ChIP analysis and 2D gel results obtained by synchronising cells in G1 and releasing them to S-phase have revealed that stalled RNAP-II complexes potentially maintain the interaction of ORCs to the selected potential ORIs during G1 phase of the cell cycle (pre-RC formation) and during the activation of pre-RC in S-phase. However, the results presented herein suggest that RNAP-II does not likely participate in origin assembly in metaphase (nocodazole). The distinct cell cycle-dependent role of RNAP-II is likely related to specific phosphorylation events that take place in different phases of the cell cycle to prevent the re-initiation of replication [73]. Cell cycle-dependent phosphatases and kinases likely arbitrate the interaction of RNAP-II molecules with Orc1p. In fact, the C-terminal domain of RNAP-II is substrate for the cell cycle kinase Cdk1p, as well as for Kin28p and Bur1p [14]. We have also recently reported that the protein phosphatase Cdc14 dephosphorylates serine residues in the CTD [74]. However, it is tempting to speculate that RNAP-II may be involved during G1 and S-phase in the time-selection of previously selected ORIs, likely to assure the timing of origin firing.

Interestingly, the chromatin interaction between IGS1-IGS2 [38] contacts two DNA replication elements; the RFB and the ARS. Conversely the Orc1p, Orc2p and Cdc6p ChIP results show that the replication proteins are only located in IGS2 (Figure 3a). However, ChIP assays were performed using low concentrations of formaldehyde to crosslink only direct DNA-protein interactions. Moreover, the protocol includes a long high-energy sonication time. Under these conditions, the indirect binding of Orc1p, Orc2p or Cdc6p to the IGS1 through RNAP-II molecules could be lost. Therefore, it cannot be dismissed that the rDNA copies, containing the IGS1–2 interaction, activate replication (See Figure S4). In the literature, there are several examples that illustrate the positive effect of chromatin interactions in DNA replication. For example, the replication origin located in the intergenic region the ß-globin gene cluster requires the locus control region (LCR) sequence that is located more than 20 kb away from the origin [75], [76]. In the case of rDNA, the absence of *RPB1* prevented IGS1-IGS2 interaction [38] and DNA replication (Figure 1 and Figure S4). In the absence of Reb1p, the RNAP-II bound strongly to the IGS1-IGS2, increasing the chromatin interactions between the two non-coding regions (Figure 6 d and e). 2D gels suggest an increase in DNA replication after growing the cells for seven generations in glucose (*REB1*) (Figure 6f). The results obtained after AM and DRB treatments likely differ from the *REB1* results because after AM treatment, the DNA loop increases [38], however, the cells did not recruit more stalled RNAP-II molecules to the chromatin [38], as occurred in the absence of Reb1p (Figure 6a). Although stalled molecules remained bound to the chromatin, and RIs were observed in 2D gels after 1 and 4 hours of transcription inhibition (Figure 2). It is important to note that we have described two DNA-loops in the rDNA. The loss of *RPB1* leads to an increase in the chromatin interaction between the promoter and enhancer of the 35S, a concomitant decrease in the IGS1–2 interaction [38] and the loss of rDNA replication (Figure 1). In contrast, the absence of Reb1p leads to an increase in the interaction between IGS1-IGS2 (Figure 6e) and a decrease in the interaction between the promoter and enhancer of the 35S gene [38]. Stalled and elongating RNAP-II complexes could differentially affect the chromatin interaction between enhancer and promoter as both DNA interactions increase after AM treatment [38]. In addition, it has been demonstrated that the upstream transcriptional activity of RNAP-I modulates rDNA replication [77]. In fact, the enhancer element located in IGS1 somehow affects replication initiation in IGS2 [77], [78]. Thus, cis- and trans- interactions between IGS1 and IGS2 regions and/or between the enhancer and promoter of the 35S gene [38], may coordinate replication timing with transcription by RNAP-I.

Cohesin loading at the CAR sequence has been reported to be dependent on RNAP-II cryptic transcripts [79]. A report published in 2010 suggested that in HeLa cells, the downregulation of cohesin limits the number of active origins by increasing the length of chromatin loops that correspond with replicon units [80]. The rDNA locus contains a binding sequence for cohesins (CAR) next to the replication origin. We have previously found that the deletion of cohesin did not decrease the DNA interaction between IGS1 and IGS2 [38]. However, rDNA is a multiple copy locus, and by using 3C methods, we can quantify the levels of IGS1-IGS2 interaction, but we cannot study the spatial organisation of the interactions [38]. Several reports claim that chromosome architecture plays a predominant role in the regulation of DNA replication origin localisation and activation, which may explain why the activation of ORIs residing at far distances imply the silence of nearby ORIs, as in the *HML* locus [25]. In the rDNA locus of *S. cerevisiae*, despite the sequence identity, only approximately 20% of the ARSs are active in any given S-phase [35], [36], [81]. Further studies will clarify the role of RNAP-II and cohesin in the spatial organisation of rDNA replication factories and recombinational processes. Although the repetitiveness of the locus makes its study difficult, it would be interesting to study the relationships among stalled RNAP-II molecules, the loading of cohesin and ARS activity in a specific rDNA copy.

The results presented herein suggest that RNAP-II is not necessary for the recruitment of ORC to the rDNA locus during metaphase (nocodazole arrest) but preserves the binding of the pre-RC to the chromatin in the G1 and S-phases. These results, together with previously published results, elicit the question of whether stalled RNAP-II complexes organise the locus into functional clusters that determine the origin timing. These findings add a new dimension to our understanding of the function of RNAP-II-associated sequences in differential origin regulation. However, other specific transcriptions factors are likely implicated in origin regulation.

## Materials and Methods

### Strains and Materials

C-terminal epitope tagging was performed using PCR allele replacement methods [11]. The yeast strains used in this study are shown in Table 1. Cells were harvested during exponential growth at different temperatures or with different sugar sources, depending of the strains used, and stored at −80°C. For each experiment, the cells were resuspended in 100-50 µl of lysis buffer and broken with glass beads in a Fast Prep machine (QBiogene) for two cycles of 20 s each with intervening incubations on ice for 5 minutes. New England Biolabs provided the *Nsp*I, *EcoR*I that were used for 3C. α-amantine (AM) and DRB were purchased from Sigma. Phospho-Ser 7 RNAP-II antibody was kindly provided by Dirk Eick. Orc1, Orc2, Cdc6, 4H8, H5 and 8GW16 antibodies were obtained from Abcam. The specificity of the RNAP-II antibodies has been studied previously [47], [48], [49]. The antibody polyclonal antiserum against a biochemically purified Reb1-LacZ was kindly provided by J.R. Warner. The J342 (P_GAL_REB1) and Z118 strains (*rpb1–1*) were kindly provided by J.R. Warner and R. Young, respectively. All values are expressed as the mean ± S.E.M. The differences between experimental groups were evaluated by using an unpaired *t*-test. All statistical calculations were performed using the GraphPad PRISM Version 5 statistical software package.

**Table 1:** Yeast Strains used in this study.

Strain	Genotype/Phenotype	Reference
W303(J47A) (P_GAL_REB1)	*MATa; ade2-1, his3–11,15, leu2–3,112, trp1–1, ura3–1, can1–100. reb1Δ::LEU2*, pBM272-41 with PGAL *REB1, HIS3*	(1) J.R. Warner (J342)
Z118 (*rpb1–1)*	*MATa; his3–200 leu2–3,112 ura3–52 ade2 rpb1–1*	[85] R. Young (Z118)
Z118 (*fob1*Δ)	*MATa; his3–200 leu2–3,112 ura3–52 ade2 rpb1–1 fob1*Δ*:: kanMX4*	This study
WT (BY4741)	*MATa; his3-1; leu2-0; met15-0; ura3-0*	Euroscarf (Y00000)
*sir2*Δ	*MATa; his3-1; leu2-0; met15-0; ura3-0 sir2*Δ*::kanMX4*	This study
*fob1*Δ (190c)	*MATa; ade2-1 ura3-1 trp1–1 leu2–3,112 his3–11 can1–100 fob1Δ::HIS3;* rDNA copy number ∼190	[86] M. Nomura (NOY1064)
*fob1*Δ (25c)	*MATa; ade2-1 ura3-1 trp1–1 leu2–3,112 his3–11 can1–100 fob1Δ::HIS3;* rDNA copy number ∼25	[86] M. Nomura (NOY1071)

(1) See [87] [85] [88] and [86] [39].

### Cell Cycle Experiments

Cells arrested in G1 were treated with alpha-factor. To release cells from the alpha-factor block, the cells were washed 2 times and transferred to fresh media containing pronase E. For releases at non-permissive temperatures, cells were exposed to 37°C in a water bath. Metaphase arrests were performed by incubating cells with nocodazole. The growth, arrest and release conditions and the concentration of drugs used for each experiment shown are explained in the text and in the figure legends.

### Neutral/Neutral Two-Dimensional Gel Electrophoresis

DNA purification and the analysis of replication intermediates by two-dimensional (2D) gel electrophoresis were performed as previously described [82]. Before the analysis, the DNA was digested with *Bgl*II, *Stu*I or *Sph*I. The RI pattern and probe used are explained in the figure legend. The first dimension was run at 1 V/cm on a 0.4% agarose gel in 1×TBE buffer for 22 h at room temperature. The second dimension was run at 5 V/cm in a 1% agarose gel in 1×TBE/0.3 µg/ml ethidium bromide for 8 h at 4°C. After electrophoresis, the gels were subjected to Southern hybridisation.

### Chromatin Immunoprecipitation

Standard ChIP was performed as previously described [38], [83]. Yeast cultures (50 ml) were grown to an OD_600_ of 0.5–0.8. The cells were crosslinked with 1% formaldehyde for 15 minutes at room temperature. Subsequently, glycine was added to 125 mM, and the mixture was centrifuged, washed with PBS and stored at −80°C. The cells were resuspended in 100 µl of IP-lysis buffer containing protease inhibitors and 1 mM PMSF and subjected to bead-beating. After recovery of the spheroplasts, the procedures were performed as described [83]. SYBr green real-time PCR was used for quantification. The primer sequences are available upon request [84]. The values are given as a percentage of material immunoprecipitated (ChIP/input).

### RNA Methods

For total RNA isolation, the cell extracts were clean from proteins and DNA using TRIZOL® Reagent (Invitrogene) before using the RNeasy Kit (Quiagen) which includes DNase treatment (RNAse-Free DNAse Set) following the manufacturer’s instructions to ensure the total degradation of DNA in the sample. ThermoScript™ RT-PCR System for First-Strand cDNA Synthesis (Invitrogene) was used. To study the total RNA levels, random primers were used for all experiments. Quantifications were performed using SYBr green real-time PCR.

### Chromatin Capturing Conformation (3C)

Chromatin was obtained as previously described for ChIP experiments without sonication. Standard 3C was performed as previously described [38].

### Whole Cell Protein Preparation for Immunoprecipitation

Exponentially growing cells (OD_600_ = 0.5, 50 ml) were washed with ice-cold PBS and resuspended in 100 µl of IP-lysis buffer containing protease inhibitors and 1 mM PMSF. The lysates were prepared by bead-beating, washed and recovered in up to 1 ml of IP buffer and sonicated in an ultrasonic bath with a Bioruptor for 10 minutes. High power outputs with short pulses were used (10 s of sonication with 20 s rest between rounds) at 4°C. The supernatant was incubated with the antibody for 15 minutes at 4°C during sonication in an ice-water bath at the lowest power outputs. After antibody incubation, protein A or protein G beads from Roche were added and incubated for 2 hours at 4°C. The beads were washed 4 times with IP buffer. The bead pellets were suspended in 80 µl of loading buffer (NuPAGE® LSD Sample Buffer [4X] from Invitrogen containing 1.42 M 2-mercaptoethanol) and boiled for 5 minutes. The supernatants were saved at −80°C for analysis by western blot. Aliquots of 10 µl were loaded in each case. For SDS–PAGE gel electrophoresis, the total cell extract was loaded onto a 4–12% or 3–8% gradient polyacrylamide gel according to NuPAGE (Invitrogen) specifications. The gels were transferred to Hybond-P membranes (Amersham-Bioscience) in Tris-glycine buffer without SDS. The membranes were blocked with 5% milk in PBS and 0.1% Tween-20 (Sigma). After probing with antibodies, the membranes were developed using ECL-Plus (Amersham).

## Supporting Information

Figure S1 **RNAP-II and Rap1p binding to rDNA.** (a) The fission yeast *S. pombe* contains multiple copies of rRNA genes in two clusters at both ends of chromosome III. The ARSs and RFBs are shown. ChIP analysis using the 4H8 antibody against RNAP-II (green line). The values are expressed as the mean, n = 2. Human ribosomal RNA genes are arranged as tandem repeats clusters at the middle of the short arms of chromosomes 13, 14, 15, 21 and 22. Human *CDC27* pseudogene and the consensus-binding site for p53 are shown. (b) ChIP analysis of Rap1p to the rDNA in mutant *rpb1–1* cells at 25°C followed by incubation at 37°C, thus inactivating RNAP-II. Rap1p was tagged with 9-MYC epitope, and anti-MYC antibody was used. The location of the primers is shown. Values are expressed as the mean. n = 2.
(TIFF)

Figure S2 **ChIP analysis of the** ***rpb1–1 ts*** **strain growing at 25°C or shifted at 37°C for 30 minutes.** Diagram of the rDNA with the locations of the replication barrier (RFBs), the replication origins (ARSs) and sites for restriction enzymes. Theoretical schemes and 2D gels of chromatin digested with *Bgl*II (a), *Stu*I (b and c) and *Sph*I (d). Results of quantification are represented in histograms (c).
(TIFF)

Figure S3 **Schematic diagram of one rDNA unit.** ChIP analysis of Ser7P-CTD and hypophosphorylated RNAP-II (8GW16) using a wild type strain within rDNA IGS regions. Mean ± S.E.M. n = 3.
(TIFF)

Figure S4 **Hypothetical model representing replication of the rDNA locus. (a)** Model representing the possible interaction between Orc1p and stalled RNAP-II complexes bound at the rDNA ARS element. The green circles represent the post-translationally modified CTD tail of the largest subunit of RNAP-II (Rpb1p). Stalled RNAP-II mediates the chromatin interaction between IGS1-IGS2 [38] by contacting two replication elements: RFB and ARS. The enhancer element located in IGS1 or other factors (X) such as Abf1p bound to the enhancer sequence [89] may be involved in modulating rDNA replication. Cis- or trans- chromatin interactions mediated by stalled RNAP-II are possibly involved in regulating origin activity.
(TIFF)
