# Supplementary figures and images for: RNAP-II Molecules Participate in the Anchoring of the ORC to rDNA Replication Origins
Source: PLoS One. 2013 Jan 4;8(1):e53405. doi: 10.1371/journal.pone.0053405 (PMC3537633; doi:10.1371/journal.pone.0053405)

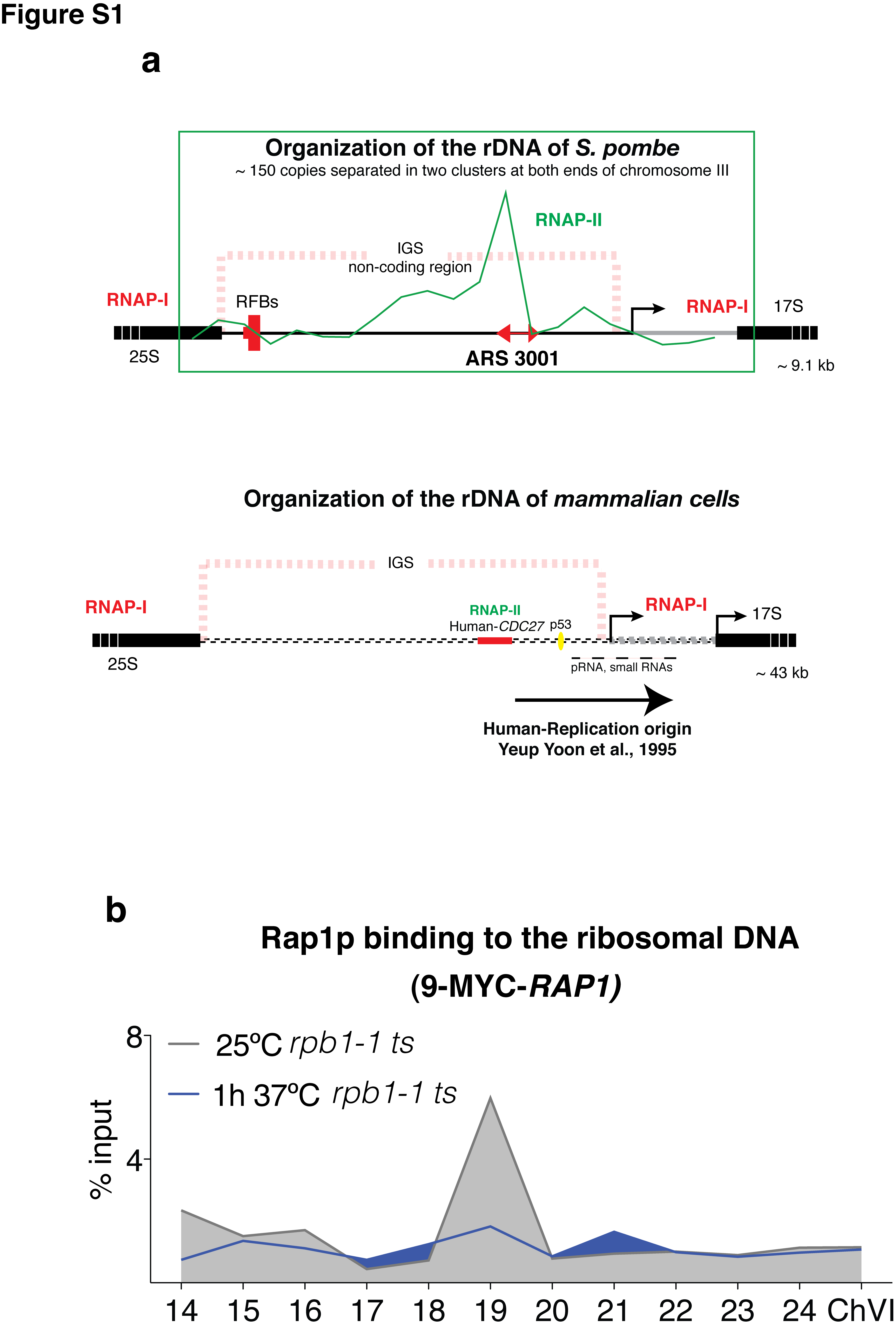

Supplement: Figure S1 — RNAP-II and Rap1p binding to rDNA. (a) The fission yeast S. pombe contains multiple copies of rRNA genes in two clusters at both ends of chromosome III. The ARSs and RFBs are shown. ChIP analysis using the 4H8 antibody against RNAP-II (green line). The values are expressed as the mean, n = 2. Human ribosomal RNA genes are arranged as tandem repeats clusters at the middle of the short arms of chromosomes 13, 14, 15, 21 and 22. Human CDC27 pseudogene and the consensus-binding site for p53 are shown. (b) ChIP analysis of Rap1p to the rDNA in mutant rpb1–1 cells at 25°C followed by incubation at 37°C, thus inactivating RNAP-II. Rap1p was tagged with 9-MYC epitope, and anti-MYC antibody was used. The location of the primers is shown. Values are expressed as the mean. n = 2. (TIFF) [file pone.0053405.s001.tiff]

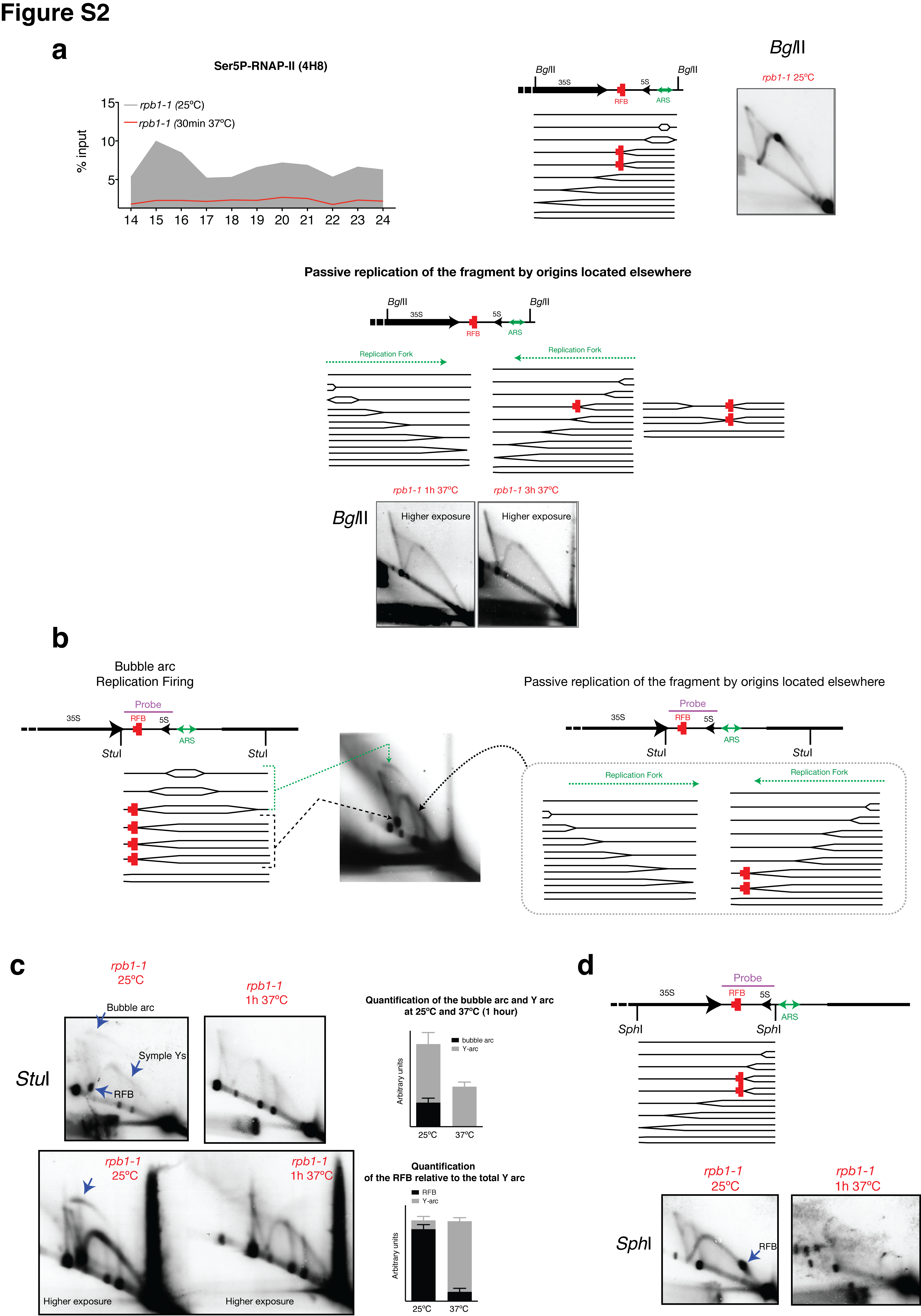

Supplement: Figure S2 — ChIP analysis of the rpb1–1 ts strain growing at 25°C or shifted at 37°C for 30 minutes. Diagram of the rDNA with the locations of the replication barrier (RFBs), the replication origins (ARSs) and sites for restriction enzymes. Theoretical schemes and 2D gels of chromatin digested with BglII (a), StuI (b and c) and SphI (d). Results of quantification are represented in histograms (c). (TIFF) [file pone.0053405.s002.tiff]

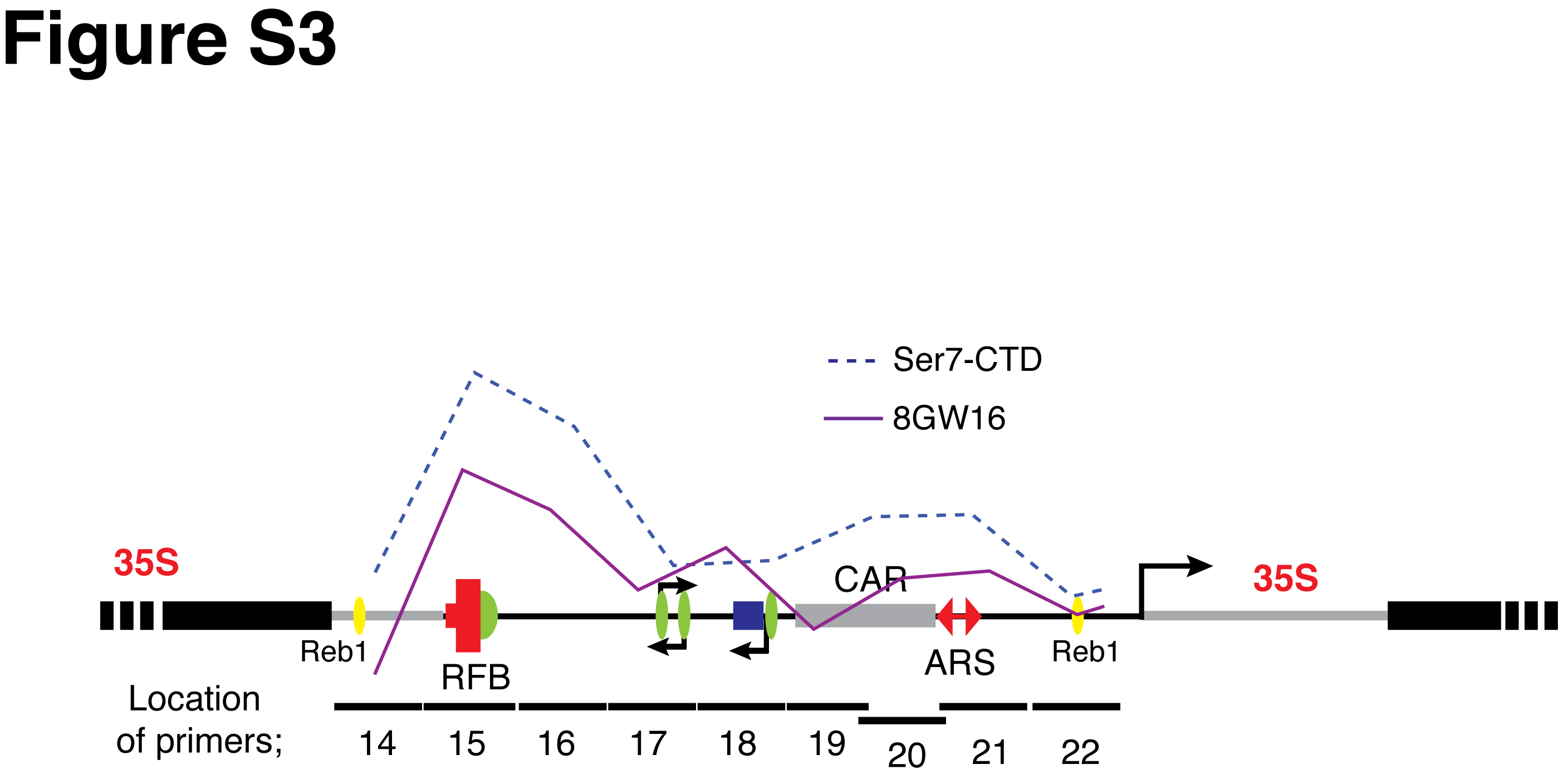

Supplement: Figure S3 — Schematic diagram of one rDNA unit. ChIP analysis of Ser7P-CTD and hypophosphorylated RNAP-II (8GW16) using a wild type strain within rDNA IGS regions. Mean ± S.E.M. n = 3. (TIFF) [file pone.0053405.s003.tiff]

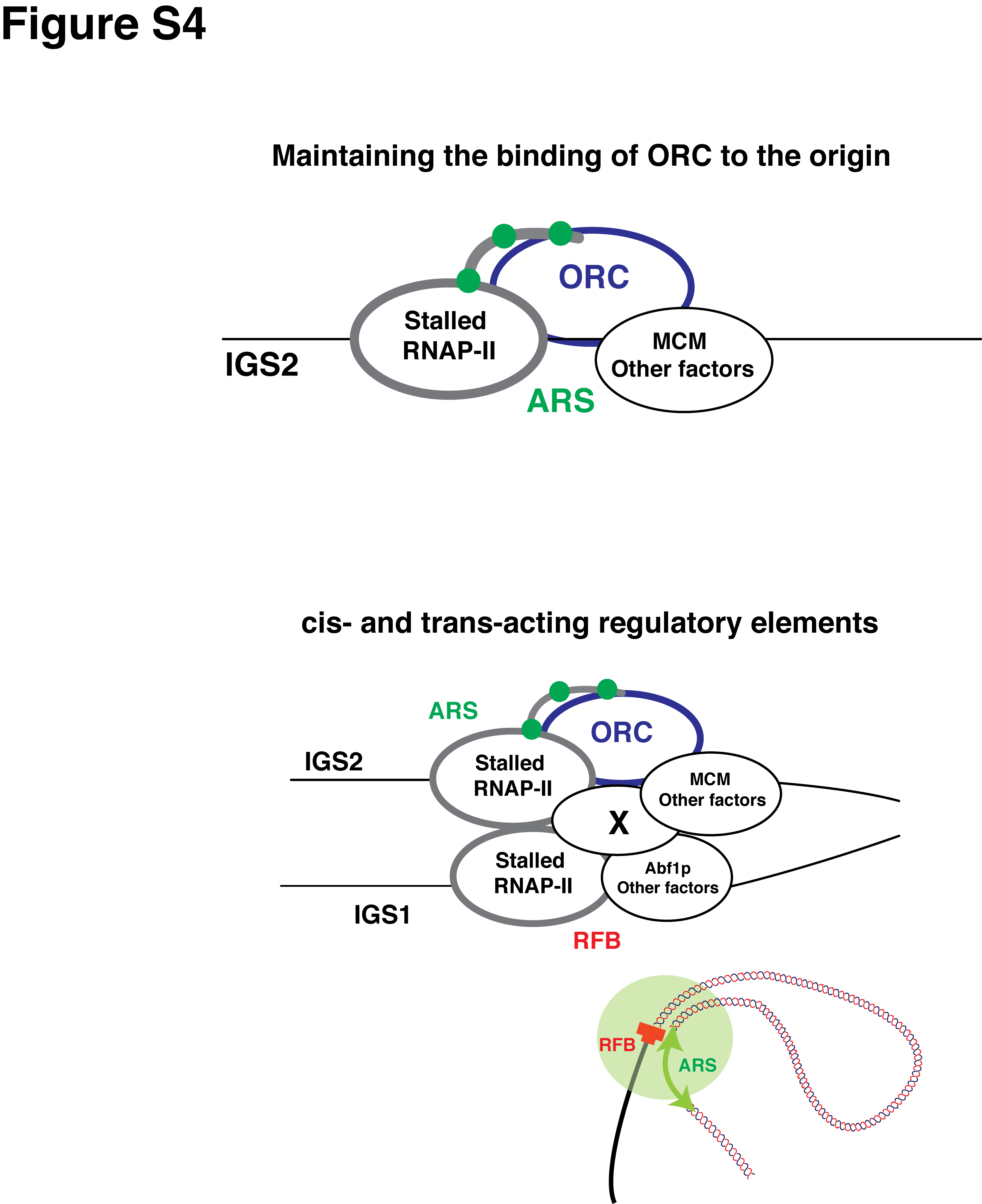

Supplement: Figure S4 — Hypothetical model representing replication of the rDNA locus. (a) Model representing the possible interaction between Orc1p and stalled RNAP-II complexes bound at the rDNA ARS element. The green circles represent the post-translationally modified CTD tail of the largest subunit of RNAP-II (Rpb1p). Stalled RNAP-II mediates the chromatin interaction between IGS1-IGS2 [38] by contacting two replication elements: RFB and ARS. The enhancer element located in IGS1 or other factors (X) such as Abf1p bound to the enhancer sequence [89] may be involved in modulating rDNA replication. Cis- or trans- chromatin interactions mediated by stalled RNAP-II are possibly involved in regulating origin activity. (TIFF) [file pone.0053405.s004.tiff]
